# Invited Commentary: The Rudimentary Right Ventricle and Achieving Pulsatile Pulmonary Blood Flow After the Björk Procedure for Tricuspid Atresia: How Little Is Too Little?

**DOI:** 10.1177/21501351231207087

**Published:** 2023-11-14

**Authors:** Ali Dodge-Khatami

**Affiliations:** Clinic for Pediatric & Congenital Heart Surgery, University Hospital RWTH Aachen, Aachen, Germany

The Björk procedure, namely the nonvalved right atrial-right ventricular (RA-RV) anastomosis using autologous pericardium,^1^ was introduced in 1978 as an alternative palliative operation for tricuspid atresia. It avoided the use of circumferential conduits or valves with their associated long-term drawbacks; otherwise inherent to modifications of an atriopulmonary Fontan connection,^2,3^ the historical standard at the time when a right outlet chamber was so small as to preclude any contribution to the pulmonary circulation.

In the initial series by Björk et al describing successful palliation in three children who were 8, 9, and 12 years of age, two had a prior classic Glenn anastomosis with severely hypoplastic right ventricles. The oldest child in this series had a large ventricular septal defect. Importantly, all three patients had normally related great vessels, adequately sized pulmonary arteries (PAs), and normal pulmonary vascular resistance.^1^ After closing the intracardiac defects and providing antegrade flow between the right atrium to the right ventricle and hence to the PAs, essentially a functional “one and a half repair with tricuspid regurgitation (TR),” or, in the absence of a prior Glenn, “biventricular physiology with free TR” was created. Björk et al hoped to draw the maximum energy potential out of RA contractility and any additional “functional” RV to provide pulmonary blood flow (PBF), avoid an intermediary valve with its inherent chronic shortcomings, and allow for septation.^1^

In a thought-provoking manuscript, Klemm et al^4^ reviewed their historical series of 66 patients who underwent a Björk procedure between 1975 and 1995. Importantly, no patient had a Glenn anastomosis prior to, or at the time of the Björk operation. Using the presence (n = 13) or absence (n = 30) of pulsatile PBF as an end point at last follow-up, they studied 43 patients who survived at least 15 years after the Björk procedure. Pulsatile PBF was defined as the difference between systolic and diastolic pulmonary artery pressures (PAPs), or Delta PAP, differing by only 3.5 mm Hg between the two groups (8.9 mm Hg = pulsatile PBF vs 5.4 mm Hg = no pulsatility). Through cardiac catheterization, cardiac magnetic resonance imaging, and exercise testing, they found higher cardiac index, higher RV end-diastolic and end-systolic volume indexes, and higher percent predicted peak oxygen consumption in patients with pulsatile PBF, compared with those without pulsatility.^4^ Interestingly but confusingly, at some point during patient follow-up when a conversion to total cavopulmonary connection (TCPC) was considered (for undescribed indications and at an unclear timing, failing Björk? symptoms? liver congestion?), nine patients underwent TCPC conversion and two patients had percutaneous valve insertion into the Björk connection when pulsatile PBF was lacking, and surgical (n = 4) or percutaneous (n = 2) valve implantation in the Björk connection was performed when pulsatile PBF was present. There were no heart transplantations and seven late deaths beyond 15 years occurred, leaving 19 patients with their original valveless Björk operation. It is unclear whether these patients are still walking around with pulsatile or nonpulsatile PBF. The authors conclude that late after the Björk procedure (unspecified if with or without a valve?), those with pulsatile PBF had larger RV volumes and better exercise performance. This could be equivalent to stating that a borderline biventricular set-up is perhaps better than optimized single ventricle physiology.

What do we do with this confusing information from precious and rare long-term survivors of an operation we no longer perform? What is the chicken and what is the egg? How little RV is too little before considering the leap from atriopulmonary or TCPC physiology toward an RA-RV connection or vice versa, and when to perform which operation? Should we still be doing the Björk procedure for borderline functional RV in tricuspid atresia and its variants and if so, should we implant valves in the RA-RV connection or not? The reader is left to choose or believe whether nature was initially kind to some patients in this series by providing enough RV mass to be incorporated into a pulsatile-flow-generating Björk, or whether pulsatile PBF as an end point leads to a larger RV and better exercise capacity.

However, the article's one large merit consists of stimulating out-of-the-box creativity in how single ventricle or near single ventricle physiology could be rethought. Given the sobering long-term results from the otherwise brilliant go-to Fontan-Kreutzer operations and our current general buzz of unhappiness leading to end-stage solutions (ie, in-Fontan propellor assist device,^5^ combined heart & liver transplantation, aggressive treatments for protein-losing enteropathy and plastic bronchitis), the article offers additional fuel to alternative pathways in attempting to deal with functional single ventricle physiology. It seems the pendulum may be swinging toward pushing the limits of not thoughtlessly performing “an unavoidable completion TCPC,” and even rerouting those extant TCPC patients back toward some other circulation, whose future still needs evaluation. These include bold staged ventricular recruitment strategies^6,7^ and steps toward biventricular conversion,^8,9^ the primary inferior vena cava-to-PA connection as an alternative staging strategy and potential destination therapy,^10^ and the ventricular switch,^11^ among others. Fontan-Kreutzer is currently still the best we have, but brave efforts by many make it seem that better is yet to come.
